# Metabolic Disease Risk in Children by Salivary Biomarker Analysis

**DOI:** 10.1371/journal.pone.0098799

**Published:** 2014-06-10

**Authors:** J. Max Goodson, Alpdogan Kantarci, Mor-Li Hartman, Gerald V. Denis, Danielle Stephens, Hatice Hasturk, Tina Yaskell, Jorel Vargas, Xiaoshan Wang, Maryann Cugini, Roula Barake, Osama Alsmadi, Sabiha Al-Mutawa, Jitendra Ariga, Pramod Soparkar, Jawad Behbehani, Kazem Behbehani, Francine Welty

**Affiliations:** 1 Department of Applied Oral Sciences, the Forsyth Research Institute, Cambridge, Massachusetts, United States of America; 2 Cancer Research Center, Boston University School of Medicine, Boston, Massachusetts, United States of America; 3 The Dasman Diabetes Institute, Kuwait City, Kuwait; 4 Ministry of Health, Kuwait City, Kuwait; 5 Faculty of Dentistry, Kuwait University, Kuwait City, Kuwait; 6 Division of Cardiology, Beth Israel Deaconess Medical Center, Boston, Massachusetts, United States of America; Wayne State University, United States of America

## Abstract

**Objective:**

The study of obesity-related metabolic syndrome or Type 2 diabetes (T2D) in children is particularly difficult because of fear of needles. We tested a non-invasive approach to study inflammatory parameters in an at-risk population of children to provide proof-of-principle for future investigations of vulnerable subjects.

**Design and Methods:**

We evaluated metabolic differences in 744, 11-year old children selected from underweight, normal healthy weight, overweight and obese categories by analyzing fasting saliva samples for 20 biomarkers. Saliva supernatants were obtained following centrifugation and used for analyses.

**Results:**

Salivary C-reactive protein (CRP) was 6 times higher, salivary insulin and leptin were 3 times higher, and adiponectin was 30% lower in obese children compared to healthy normal weight children (all *P*<0.0001). Categorical analysis suggested that there might be three types of obesity in children. Distinctly inflammatory characteristics appeared in 76% of obese children while in 13%, salivary insulin was high but not associated with inflammatory mediators. The remaining 11% of obese children had high insulin and reduced adiponectin. Forty percent of the non-obese children were found in groups which, based on biomarker characteristics, may be at risk for becoming obese.

**Conclusions:**

Significantly altered levels of salivary biomarkers in obese children from a high-risk population, suggest the potential for developing non-invasive screening procedures to identify T2D-vulnerable individuals and a means to test preventative strategies.

## Introduction

The prevalence of pediatric obesity has increased worldwide in recent years and raised urgent concern about expected metabolic dysregulation and serious comorbidities, such as T2D, likely to arise among obese children as they age into adulthood [1]. The relationship is unknown among adult-onset, obesity-associated pathologies and exposure of these individuals to diet-induced obesity while still children. Furthermore, it is not known if at-risk cohorts of pediatric subjects can be identified by non-invasive measures early enough during development of pediatric obesity for effective interventions to reduce risk of T2D. The importance of the answers to these questions is widely recognized, thus countries with significant prevalence of pediatric obesity are devoting resources to understand the scope of this problem and devise appropriate public health recommendations.

In this research, we describe an approach that uses saliva samples as a basis for diagnosis. The selection of biomarkers for evaluation in this study of obese children was focused on adipokines (adipocytokines), because concentrations of these factors partly define metabolic health. A subset of these proteins is of particular interest for immunometabolism, including adiponectin, leptin and resistin, which are adipocyte-derived hormones secreted in saliva [2], salivary [3] monocyte chemotactic protein-1 (MCP-1) and salivary [4], [5] tumor necrosis factor-alpha (TNF-α). Interleukins (IL) comprise a second major class of investigated biomarkers, each of which has also been measured in saliva, but not in an obese adolescent population. Specifically, IL-1β is associated with periodontal inflammation [6]; IL-6 has been measured in periodontitis patients; IL-4, IL-10, IL-12 and IL-17 are related to Sjogren’s syndrome [7]; IL-10 is reduced in periodontitis patients [5]; IL-8 is related to dental caries in adolescents [4] and oral cancer [8]; IL-13 is identified in the sputum of asthmatics [9]; IL-17 is lower in patients with periodontal disease [10]; and interferon γ (IFN-γ) is higher in the saliva of control subjects without Sjogren’s syndrome [7]. Thus, adipokines and cytokines from sources other than peripheral blood have been assayed and associated with disease states. Both salivary immunoreactive insulin [11] and salivary ghrelin [12] have been associated with T2D and obesity, and were included in our analysis. Two oral inflammatory disease markers were also included, matrix metalloproteinase-9 (MMP-9) and myeloperoxidase (MPO). MMP-9 is a protease often found elevated in patients with periodontal disease [13] and oral cancer [14]; whereas MPO, a peroxidase often used as a measure of neutrophil degranulation, is elevated in diabetic patients [15]. Vascular endothelial growth factor (VEGF) was included because it appears in saliva of diabetic women [16]. C-reactive protein (CRP) was included because it is associated with inflammation and cardiovascular disease [17]. Collectively, the panel defines inflammatory and metabolic processes important for metabolic health in obesity.

Blood elements that partition into saliva have attracted interest [18]. Saliva has major advantages over peripheral blood for the study of disease development in childhood because sample collection is non-invasive. Here, we collected 8,319 saliva samples from 10–12-year old Kuwaiti children, a high risk population for obesity and T2D development [19]. We measured salivary concentrations of 20 hormones and cytokines in a random subset of 744 subjects. The well-established relationship between insulin-resistant obesity and systemic elevation of pro-inflammatory cytokines [20] supports our hypothesis that the saliva of obese children shows informative imbalances in critical immunometabolic factors [21]. The results of this study provide insight into the early development of metabolic disease in children and establish that non-invasive methods are robust and useful for data collection in studies of vulnerable subjects.

## Materials and Methods

### Subject Selection

The validation study for US subjects was approved by the Forsyth Institutional Review Board in Cambridge MA, U.S.A. The study of Kuwaiti subjects (4^th^ or 5^th^ grades) was approved by the Dasman Diabetes Institute Ethical Review Committee in Kuwait. Arabic language informed consent was signed by parents/guardians in advance. Subject assent was obtained the day of the visit.

### Data Collection

Data and saliva samples were collected from 8,319 subjects during 182 visits to 39 Kuwaiti schools between October 2, 2011 and May 15, 2012. Subject identification, height, weight, blood pressure, food preferences, oral examination, fitness and sleep parameters were entered into a programmed iPad (Apple, Cupertino, CA) system for internet transfer. Fitness was measured by heart rate elevation (beats/minute) following a standard 3-minute exercise [22]. Body weight categories were defined using a Body Mass Index (BMI) z-score [23]. Blood and saliva samples were collected for insulin analysis from February 23–September 23, 2011 from 53 US subjects living in Maine and Massachusetts. Otherwise, the US and Kuwait protocols were identical.

### Saliva Collection

After a 15 ml water rinse of the mouth, whole saliva was collected by expectoration of approximately 3 ml into a sterile tube kept on ice. Samples were collected starting at 8∶30 AM before the subjects had breakfast. The time of start and end of collection was recorded for all children. Iced samples were immediately transported to the Dasman Institute where they were weighed to determine volume collected and centrifuged to remove particulate debris and exfoliated mucosal cells at 2,800 RPM for 20 minutes at 4°C. Supernatant aliquots were transferred to screw-cap 2D barcoded storage tubes (Thermo Scientific) read by a barcode reader (Thermo Scientific VisionMate ST). The barcode was captured with subject number to spreadsheet. The sample vials were sealed by a torque-controlled tube capper (Thermo Scientific 8-Channel Screw Cap Tube Capper), placed in a 96-vial rack (Thermo Scientific Latch Rack) and frozen at −80°C. Racks were air-transferred from Kuwait under temperature-monitored dry ice (Biocair, Boston MA) to the Forsyth Institute and maintained at −80°C until assay (average time to assay = 0.88±0.06 y).

From the 8,319 samples, 744 samples were selected for assay such that 93 were from subjects of each of the defined body weight categories described in the data analysis section for each sex. Random selection was achieved by assigning a random number between 1 and x = integer (total number in each group/93) to each subject, sorting for all assigned the number 1 and selecting the first 93. All body weight categories included 186 subjects evenly distributed by sex except for underweight subjects, where, due to a shortage of male underweight subjects, 64 male and 122 female subjects were selected.

### Multiplex Analysis of Salivary Markers

All assays (744 saliva samples for 20 biomarkers) were performed on 25 µl of saliva supernatant using four multiplex magnetic bead panels on a Luminex 200 platform (Luminex, Austin, TX). Results were evaluated using Bio-Plex Manager (Version 5.0; Bio-Rad, Hercules, CA). IFN-γ, IL-10, interleukin-12p70 (IL-12P70), IL-13, IL-17A, IL-1β, IL-4, IL-6, IL-8, MCP-1, TNF-α and VEGF were measured by a 12-plex human cytokine/chemokine panel with no dilution (Millipore cat #HCYTOMAG-60K; lot #2055690). Metabolic hormones ghrelin, insulin and leptin were measured by a 3-plex human metabolic hormone panel with no sample dilution (Millipore cat #HMHMAG-34AK; lot #2055724). MPO and MMP-9 were measured by a 2-plex human cardiovascular disease panel with 1∶2 sample dilution (Millipore cat #HCVD1–67AK; lot #2055723). Adiponectin (total), CRP and resistin were measured in a 3-plex human obesity panel with 1∶2 sample dilution (R&D cat #LOB000, LOB1065, LOB1707, LOB1359; lot #300710). The panel used to evaluate insulin in blood and saliva of US subjects was a human metabolic hormone panel single plex for insulin (Millipore cat #HMHMAG-34K-01EMD) with no dilution. All assays were performed following manufacturers’ protocol, with the exception of an additional 3 standards to increase the range of detection [24].

Saliva supernatants were thawed at 4**°**C overnight and kept on ice throughout the assay procedures. Briefly, all kit components were brought to room temperature and reagents were prepared as instructed (wash buffers, beads, standards, etc.). Assay plates (96-well) with assay buffer, standards, samples and beads were covered and incubated on plate shaker (500 rpm) for either 3 hours at room temperature for the obesity panel, or overnight at 4**°**C. Plates were washed and detection antibody cocktail added. Plates were covered and incubated at room temperature for 1 hour on a plate shaker. Streptavidin-phycoerythrin fluorescent reporter was added to all wells, and the covered plate incubated for 30 minutes at room temperature on a shaker. Plates were washed three times and beads resuspended in wash buffer, placed on shaker for 5 minutes, read and evaluated.

### Salivary Protein Assay

A subset of 213 saliva supernatants (approximately 54 from each body weight category) were randomly selected and assayed for total protein. The assay was by a commercial kit (Pierce 660 nm protein assay, Pierce Biotechnology Rockford, IL) which has a manufacturer’s stated working range of 50–2,000 µg/ml. Values were determined on 10 µl saliva supernatant samples using bovine serum albumin as a standard implemented on a robotic chemical analysis unit (EVO 100 with a Sunrise spectrophotometer set at 660 nm, Tecan US, Morrisvile NC).

### Data Analysis

Body weight categories were defined on BMI z-score based on published growth charts [23]. By this criterion, obese was ≥95^th^ percentile, overweight was ≥85^th^−95^th^ percentile, normal healthy weight was ≥5th and 85^th^ percentile and underweight was <5^th^ percentile [25]. Salivary flow rate was computed from the start time, stop time and tarred salivary weight. To account for the non-normality of multiprobe data, a multivariate rank-based Wilcoxon regression method [26] was applied. The relationship between the probe concentration and body weight categories were adjusted for age and sex. Values for age, BMI, waist circumference (WC), systolic and diastolic blood pressure, and fitness were analyzed for significance between body weight categories by a Kruskal-Wallis rank sum test followed by pairwise comparisons using the Wilcoxon rank sum test. Predictor variables, diagnostic sensitivity and specificity were determined by CART software (Salford Systems, San Diego, CA). Log transformation of both insulin measurements preceded their analysis.

## Results

Saliva samples (744) were randomly selected from the 8,319 total to provide 186 for each category of underweight, normal healthy weight, overweight and obese. Characteristics of these groups are evaluated in Table 1. No significant differences in age between groups were noted. BMI, WC, blood pressure and fitness exhibited significant incremental increases as the body weight category moved to obese. In every body weight category, BMI and WC significantly differed from each other. Systolic and diastolic blood pressures were significantly different only between normal and overweight or obese subjects. Obese subjects had significantly lower fitness as measured by exercise stimulated heart rate elevation than any other category. Obese subjects had 64% higher BMI, 40% greater WC, 17% higher systolic and diastolic blood pressure and 50% greater exercise elevation of heart rate as a measure of unfitness compared to normal weight subjects. Table S1 in File S1 shows similar statistics from 53 US subjects used to measure the relative concentration of insulin in saliva and plasma.

**Table 1:** Age, BMI, waist circumference, systolic blood pressure, diastolic blood pressure and fitness of 744 Kuwaiti children (M = Male, F = Female).

	Probe	Age		BMI		Waist		Systolic BP		Diastolic BP		Fitness	
	Units	years	p	kg/m^2^	p	cm	p	mmHg	p	mmHg	p	beats/min.	p
		(mean±S.D.)		(mean±S.D.)		(mean±S.D.)		(mean±S.D.)		(mean±S.D.)		(mean±S.D.)	
**1. Underweight**	**M(64)**	11.76±0.43	NS	14.13±0.64	*2,3,4	60.48±54.10	*2,3,4	97.18±10.75	*3,4	67.25±10.95	*3,4	18.81±17.24	*4
**(<5th percentile)**	**F(122)**	11.51±0.58		13.86±0.71		54.90±6.03		100.92±14.85		68.66±12.43		22.77±19.43	
**2. Normal**	**M(93)**	11.52±0.62	NS	17.46±1.50	*1,3,4	58.50±4.66	*1,3,4	100.53±12.92	*3,4	68.72±12.01	*3,4	18.06±18.09	*4
**(≥5^th^–85th percentile)**	**F(93)**	11.39±0.59		17.73±1.88		61.64±6.29		104.54±15.26		70.74±11.15		26.58±23.99	
**3. Overweight**	**M(93)**	11.55±0.56	NS	22.12±0.97	*1,2,4	70.19±5.11	*1,2,4	112.74±13.51	*1,2,4	74.94±11.49	*1,2,4	23.70±18.48	*4
**(≥85th−95th percentile)**	**F(93)**	11.44±0.58		22.86±1.10		72.59±5.67		114.47±13.65		77.23±12.98		24.68±19.86	
**4. Obese**	**M(93)**	11.50±0.50	NS	28.79±4.14	*1,2,3	83.49±9.47	*1,2,3	122.37±18.24	*1,2,3	82.18±14.09	*1,2,3	31.89±21.82	*1,2,3
**(≥95th percentile)**	**F(93)**	11.43±0.57		28.80±3.62		84.69±8.06		118.22±14.49		81.39±13.63		35.17±19.47	

Significance tested as pooled male and female subjects in each body weight category for p<0.001 (the Bonferonni correction for 36 comparisons). NS, not significant when compared with any other group. *, significant (p<0.001) differences between the category and the numbers representing other categories.

*significantly different p<0.002.

The assay performance is summarized in Table S2 in File S1. The median concentration of 17 biomarkers was greater than the manufacturer’s stated assay sensitivity. Median values fell below the assay sensitivity for IL-10, leptin and ghrelin. The analysis software allowed extrapolation beyond the lowest standard, provided the value did not fall on or below the blank. Using this feature, although the concentration cannot be said to have been accurately measured, the assay provided a means to order low concentrations for non-parametric rank analysis. All probes provided non-zero values for 95 to 100% of the samples tested except for leptin; 37.4% of the leptin determinations were too low to measure. In the leptin analysis, however, the percentage of measurable samples increased with increasing obesity so that analysis by non-parametric rank could be performed without introducing bias.

**Figure 1:**
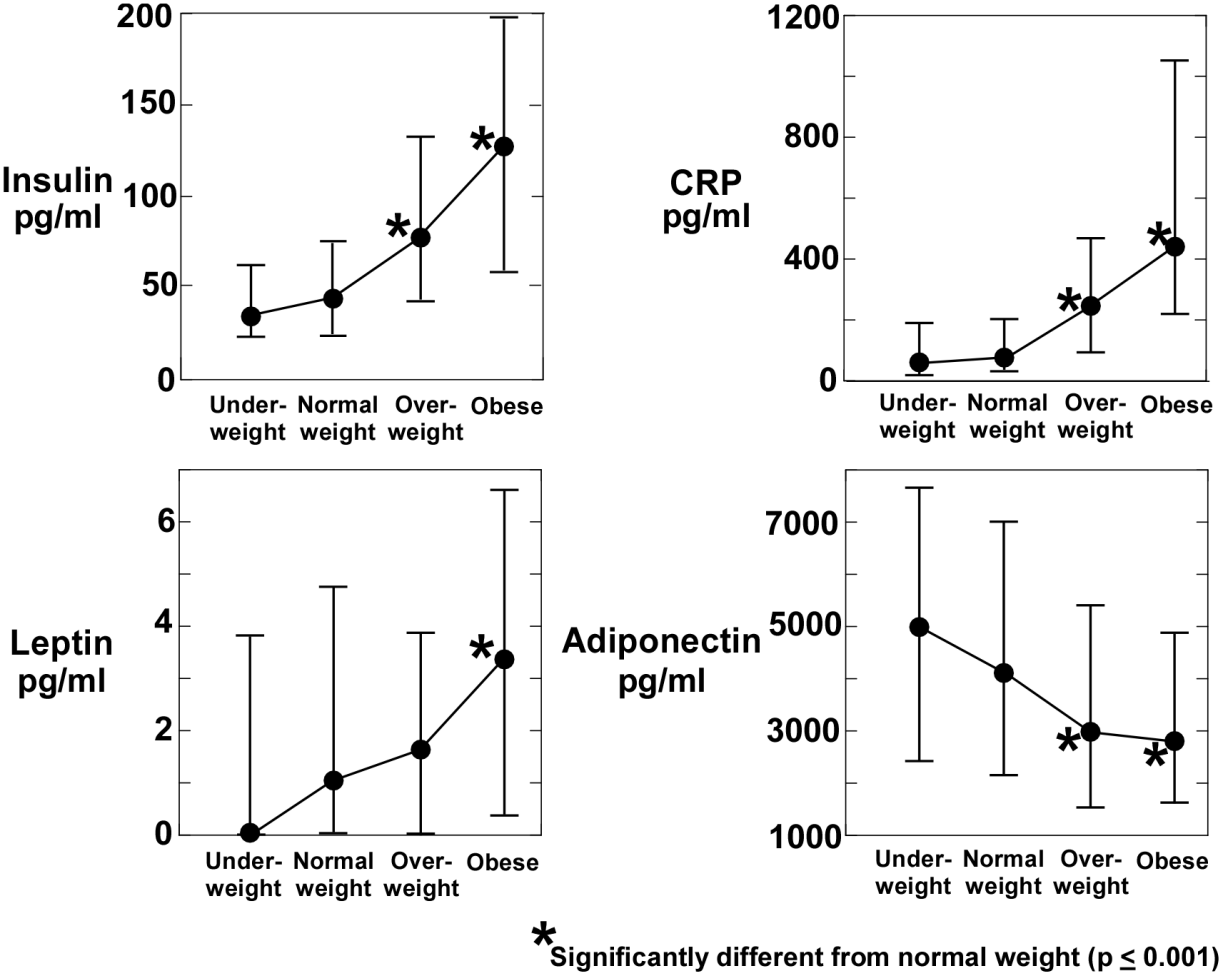
The concentration of biomarkers in saliva supernatant that were significantly different in obese children compared to normal weight children. Insulin, CRP and leptin significantly increased with obesity whereas adiponectin decreased. Central values represent the median concentration and whiskers represent the interquartile range (+75^th^ percentile, −25^th^ percentile).

The assay results are summarized in Figure 1 and Table 2. Of the 20 probes tested, significant differences between obese and normal weight subjects occurred in the concentration of insulin, CRP, adiponectin and leptin. CRP and insulin were significantly elevated in obese relative to normal subjects. Salivary insulin levels in obese subjects (median = 127 pg/ml) were almost 3 times that of normal weight subjects (median = 44 pg/ml) and salivary CRP of obese subjects (median = 435 pg/ml) was almost 6 times that of normal weight subjects (median = 76 pg/ml). The estimated concentration of salivary leptin in obese subjects (median = 3.3 pg/ml) was 3 times that of normal weight subjects (1 pg/ml). Salivary adiponectin decreased by approximately 30% with increasing obesity from that of normal healthy weight subjects (median 4,083 pg/ml) to overweight and obese subjects (median = 2981 and 2798 pg/ml respectively). Differences between normal and obese subjects for insulin, CRP, adiponectin and leptin were all highly significant (p<0.0001). There were no significant differences between biomarker concentrations of underweight and normal healthy weight subjects. Salivary levels of the remaining 16 probes (Figure S1, Table S3 in File S1) did not significantly change when analyzed by body weight categories.

**Table 2:** Concentration of insulin, C-reactive protein (CRP), adiponectin and leptin in the saliva supernatant of 744 children by body weight category and sex.

	Probe	Insulin	CRP	Adiponectin	Leptin
	Parameter	(median, intraquartile range)	(median, intraquartilerange)	(median, intraquartile range)	(median, intraquartile range)
	Units	pg/ml saliva	pg/ml saliva	pg/ml saliva	pg/ml saliva
**Underweight** **(186)**	**M(64)**	39.17, 48.63	56.64, 92.32	4,421, 6,424	0.01, 5.31
	**F(122)**	34.30, 38.02	61.99, 182.74	5,060, 4,573	0.01, 3.22
**Normal (186)**	**M(93)**	39.39, 45.38	73.01, 153.75	4,220, 5,303	1.06, 4.77
	**F(93)**	44.70, 54.38	77.15, 186.95	3,994, 5,052	0.63, 4.61
**Overweight** **(186)**	**M(93)**	80.39, 88.74	177.46, 311.93	2,402, 3,785	1.06, 3.26
	**F(93)**	76.25, 87.13	281.39, 516.54	3,322, 3,693	2.41, 5.03
**Obese (186)**	**M(93)**	112.98, 125.09	429.44, 668.52	2,548, 2,779	3.16, 6.40
	**F(93)**	143.50, 150.24	443.13, 1033.29	3,062, 3,752	3.70, 6.41
**Wilcoxon regression p**	**Age**	0.107	0.083	0.373	1.000
	**Sex**	0.337	0.028	0.471	1.000
	**Overweight**	<0.0001	<0.0001	0.001	0.408
	**Obese**	<0.0001	<0.0001	<0.0001	<0.0001
	**Underweight**	0.157	0.266	0.142	1.000

Summary statistics are median, interquartile range (N subjects). Values recorded for sixteen additional probes tested are in Table S3 in File S1. Probability levels for age, sex, overweight, obese and underweight were computed by Wilcoxon regression relative to normal healthy weight children.

**Figure 2:**
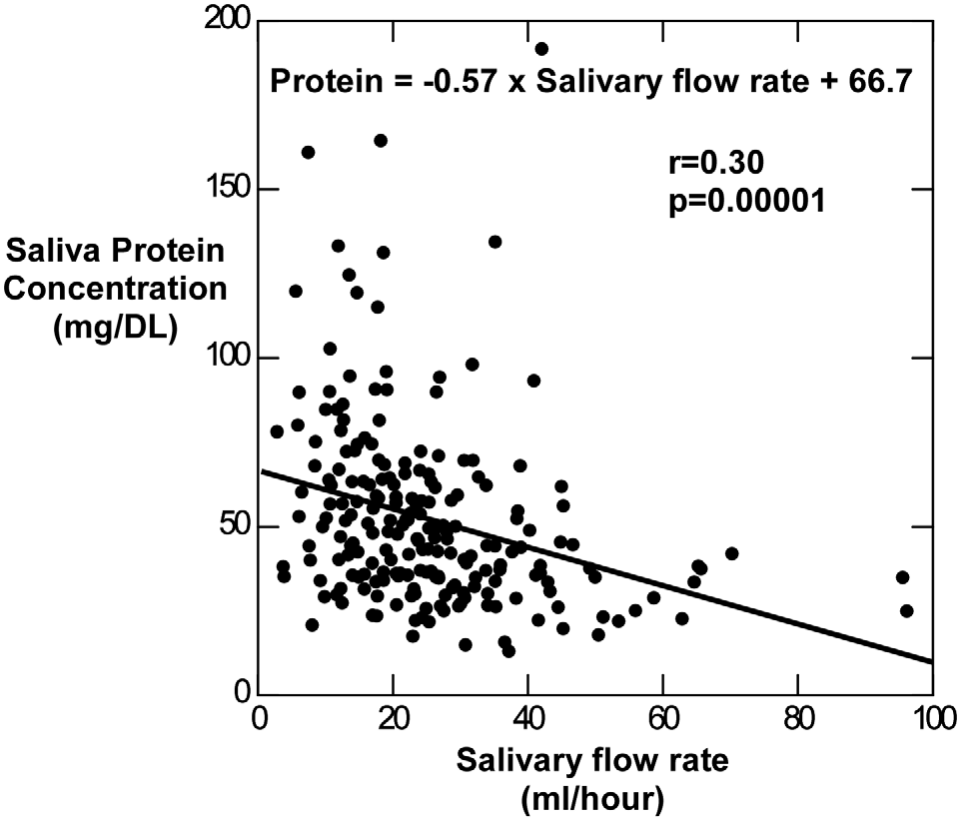
Total salivary protein concentration and salivary flow rate. In the subset of 213 samples from children measured for total salivary protein, protein content significantly declined with increased salivary flow rate.

**Figure 3:**
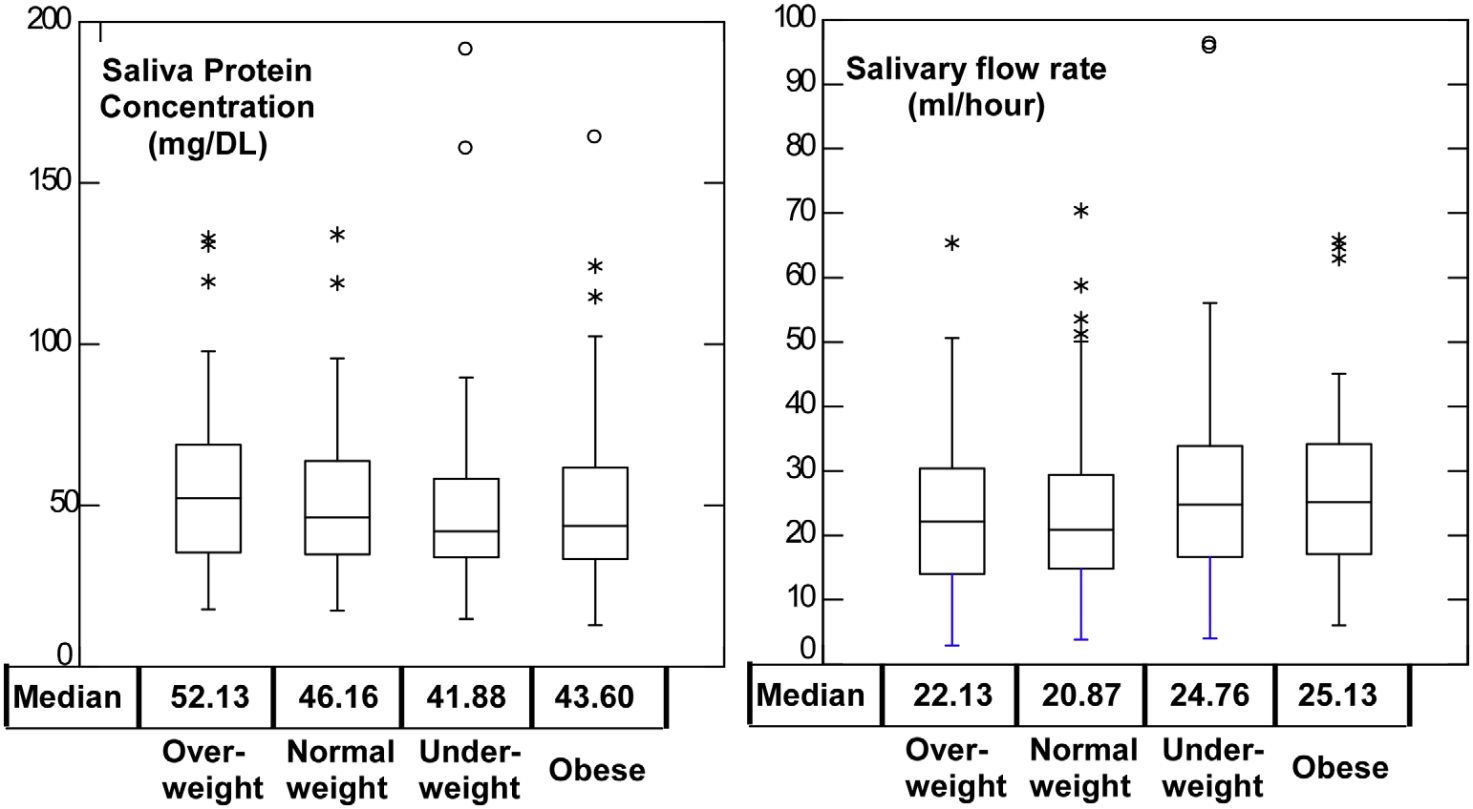
Salivary protein concentration and salivary flow rate of children by body weight category. Neither protein content nor salivary flow rate differed significantly in this subset of 213 samples between children in each body weight category.

Evaluation of saliva supernatants normalized by salivary total protein content (Table 3), indicated that significant levels of biomarkers obtained were similar to those measured using salivary concentration. Salivary protein concentration significantly decreased with increasing salivary flow rate (Figure 2). Neither salivary protein concentration nor salivary flow rate varied significantly between body weight categories (Figure 3).

**Table 3:** Concentration of insulin, CRP, adiponectin and leptin normalized by total protein of a subset of 213 randomly selected children.

	Probe	Insulin	CRP	Adiponectin	Leptin
	Parameter	(median, intraquartile range)	(median, intraquartile range)	(median, intraquartile range)	(median, intraquartile range)
	Units	pg/ml saliva	pg/ml saliva	pg/ml saliva	pg/ml saliva
**Underweight (27)**	**M(12)**	39.11, 84.01	61.66, 143.95	13, 363, 13, 812	0.02, 4.47
	**F(15)**	87.84, 83.83	117.67, 217.16	8, 168, 6, 591	0.03, 4.66
**Normal (71)**	**M(36)**	86.45, 133.87	142.82, 236.09	8, 919, 7, 590	1.04, 7.65
	**F(35)**	53.56, 83.21	180.74, 327.00	11, 426, 10, 123	0.67, 8.59
**Overweight (57)**	**M(19)**	192.29, 208.82	430.76, 625.88	7, 152, 5, 916	5.14, 5.82
	**F(38)**	168.09, 257.48	607.19, 787.82	9, 184, 6, 897	3.38, 8.71
**Obese (58)**	**M(27)**	211.83, 203.68	1, 038.31, 1, 484.94	6, 337, 3, 550	3.39, 12.44
	**F(31)**	188.95, 236.98	828.61, 1, 560.40	7, 740, 5, 233	4.77, 11.75
	**Age**	0.047 (0.038)	0.825 (0.947)	0.080 (0.097)	0.970 (1.000)
**Wilcoxon regression p**	**Sex**	0.452 (0.619)	0.564 (0.253)	0.181 (0.329)	0.970 (1.000)
mg protein (ml saliva)	**Overweight**	0.262 (0.448)	0.132 (0.266)	0.560 (0.911)	0.018 (0.020)
	**Obese**	<0.0001 (<0.0001)	<0.0001 (<0.0001)	0.082 (0.030)	0.017 (0.020)
	**Underweight**	<0.0001 (<0.0001)	<0.0001 (<0.0001)	0.028 (0.017)	0.003 (0.012)

Probability levels for protein-normalized saliva samples for age, sex, overweight, obese and underweight were computed by Wilcoxon regression relative to normal healthy weight children are listed with probability levels of the subset based on salivary concentration in parentheses.

**Figure 4:**
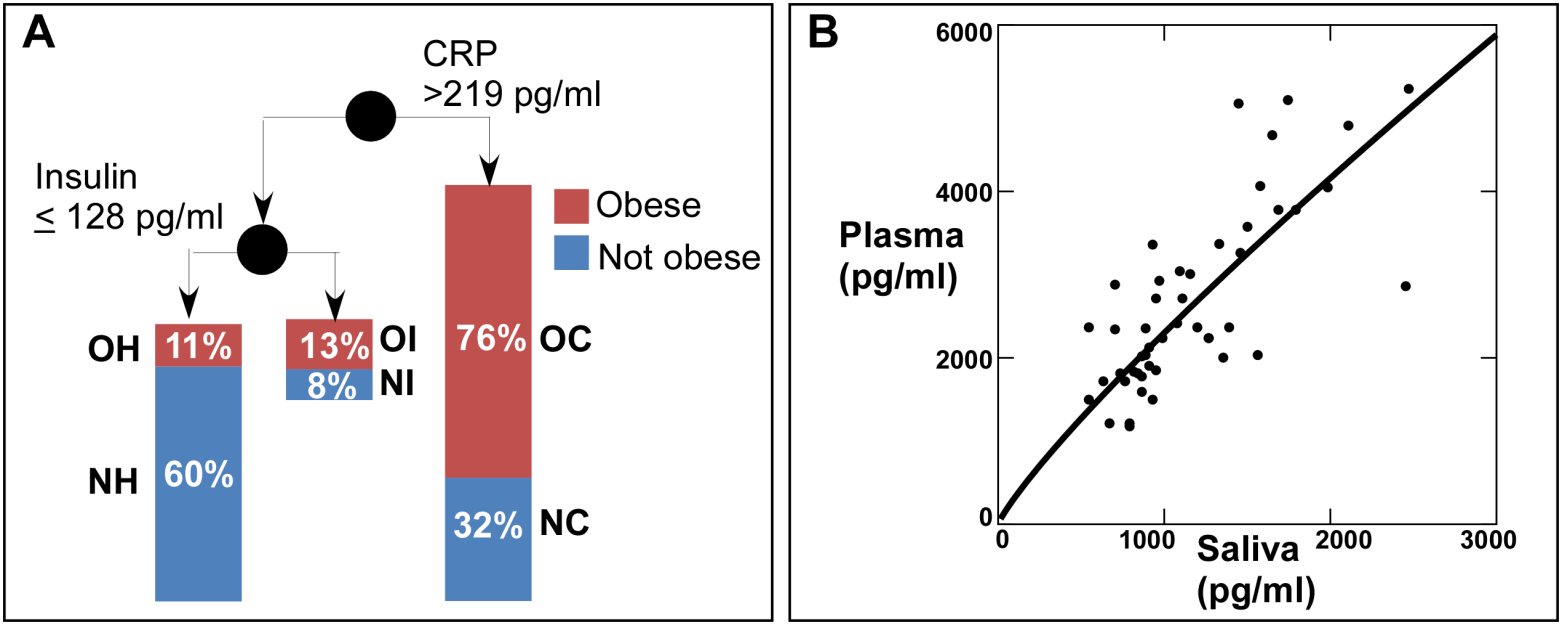
A: Categorical decision tree describing identification of children that are obese or not obese. 76% of the obese children were identified by the predictor variable salivary CRP >219 pg/ml. Of the lower salivary CRP saliva samples, 13% of the obese children were identified by the predictor variable insulin >128 pg/ml and 11% had insulin ≤128 pg/ml. B: Insulin concentration in fasting saliva supernatant and plasma of 53 adolescent donors. These data were fitted after log transformation to obtain the equation Ln(Plasma) = 0.85*Ln(Saliva) +1.84 with r^2^ = 0.67. Based on this approximation, the predictor variable of 128 pg/ml saliva insulin would be approximately 67 pmoles/L of plasma insulin (or 11 µU/ml using the conversion factor 1µU/ml = 6.00 pmol/L).

The diagnostic implications of these findings were evaluated by classification tree topology (Figure 4A). Of the obese subjects, 76.3% had >219 pg/ml CRP in their saliva, indicating that the inflammatory state was the most common form of obesity. Of the obese subjects with lower levels of CRP (<219 pg/ml), 13% had high insulin (>128 pg/ml) and 11% had low insulin. The obese group with CRP <219 and insulin <128 would be predicted to be ‘metabolically healthy’. Using only salivary CRP and insulin as predictors, the overall diagnostic sensitivity for identifying obesity by biomarker status was 89% and specificity was 61%.

We used Luminex analysis to evaluate correspondence between saliva and plasma concentration of insulin in a group of 53 American subjects similar in age to the Kuwaiti subjects (Figure 4B). We found that the concentration of immunoreactive saliva insulin is approximately half of the plasma concentration and estimates of plasma insulin with high correlation (r^2^ = 0.67). By this approximation, the predictor variable of 128 pg/ml salivary insulin is equivalent to 68 pmoles/L = 11 µU/ml in plasma, a value higher than that reported for normal fasting subjects 38–46 pmoles/L = 6.3–7.6 µU/ml [27].

Investigation of the properties of each of the six groups described by this categorization tree revealed significant differences relative to the normal healthy (NH) group in both clinical measures and biomarker concentrations (Table 4). Systolic blood pressure, BMI, WC and insulin were significantly elevated in all categories except for the non-obese healthy group (NH). Diastolic blood pressure was significantly elevated in the obese with high insulin (OI) and obese with high CRP (OC) groups. Low fitness was significantly increased in the OC group. CRP and IL-6 were significantly elevated in both high CRP groups (OC and NC) and leptin was significantly elevated in the obese high CRP group (OC). The non-obese, high CRP group (NC) exhibited elevation of multiple biomarkers (IL-10, resistin, IL-1β and MMP-9). The obese but healthy group (OH) exhibited significantly lower concentrations of IL-10 and adiponectin.

**Table 4:** Median values for measures and biomarkers of groups defined by CRP and insulin concentration in saliva supernatant.

	Non-inflammed				Inflammed	
	CRP ≤219				CRP >219	
	Insulin ≤128		Insulin >128			
Clinical Measure	OH	NH	OI	NI	OC	NC
BMI (Kg/M^2^)	**26.4** ***	16.2	**29.8** ***	**21.4** ***	**28.2** ***	**21.1** ***
Waist circumference (cm)	**78.7** ***	58.4	**86.4** ***	**68.6** ***	**83.8** ***	**66.0** ***
Systolic BP (mmHg)	**121** ***	101	**122** ***	113**	**120** ***	**109** ***
Diastolic BP (mmHg)	78*	70	**84** ***	75*	**79** ***	72.0
Fitness (beats/min)	34.8**	19.5	29.5	20.0	**35.5** ***	23.0
N	20	337	24	44	142	177
% of total	2.7	45.3	3.2	5.9	19.1	23.8
% of obese	10.8		12.9		76.3	
	**CRP ≤219**				**CRP >219**	
	**Insulin ≤128**		**Insulin >128**			
**Biomarker**	**OH**	**NH**	**OI**	**NI**	**OC**	**NC**
CRP (pg/ml)	62.3	54.1	126.6**	67.1	**604.6** ***	**521.0** ***
Insulin (pg/ml)	**74.5** ***	41.8	**186.8** ***	**177.4** ***	**121.5** ***	**54.3** ***
IL-6 (pg/ml)	3.8	6.2	3.4*	4.6	**8.4** ***	**10.7** ***
Leptin (pg/ml)	1.3	0.6	5.7**	0.5	**3.3** ***	1.4*
IL-10 (pg/ml)	**2.8** ***	4.3	2.7*	2.8*	5.5*	**6.1** ***
Adiponectin (pg/ml)	**1201** ***	3582	1872**	1788**	3134	4816**
Resistin (pg/ml)	1073*	1555	1116	948*	1812	**2296** ***
IL-1β (pg/ml)	24.5	41.9	16.4*	24.7*	47.7	**77.6** ***
MMP-9 (pg/ml)	8.9*	17.1	8.3*	12.8	22.7*	**27.5** ***
IL-8 (pg/ml)	260.5*	440.8	243.4*	259.4*	476.7	644.8**
TNF-α (pg/ml)	4.3*	5.8	1.8**	3.1**	6.6	7.9**
VEGF (pg/ml)	206.5*	265.3	308.4	348.8**	290.5*	317.7*
IL-12P70 (pg/ml)	13.2	14.7	8.2	9.3	9.9	17.5*
IL-17A (pg/ml)	1.6	1.0	0*	0.2*	0.7	1.5*
MCP-1 (pg/ml)	174.1*	236.8	282.3	308.3*	267.0*	259.8
Ghrelin (pg/ml)	1.9	1.1	1.9	1.1	1.1	1.4
IFNγ (pg/ml)	3.0	2.2	1.0*	1.3	1.9	2.6
IL-13 (pg/ml)	6.1	6.9	3.0*	4.5*	6.7	7.1
MPO (pg/ml)	26.3	30.7	25.5	32.5	30.5	31.7
IL-4 (pg/ml)	7.7	7.5	5.2	5.8	7.2	8.8

Categorical comparisons were by the Mann-Whitney U test. A Bonferroni correction of 0.0004 (for overall p<0.05) was applied to control the familywise error rate associated with 125 test procedures (in boldface).

***p<0.0004,

**p<0.001,

*p<0.05.

## Discussion

Many blood factors also occur in saliva and their levels often correlate. In our study, we surveyed 20 possible biomarkers related to obesity and found four that exhibit significant change with increasing body weight in a pediatric population. These data suggest that saliva could be a useful blood surrogate for the study of metabolic complications of obesity in children, where repeated blood sampling can be both traumatic and difficult.

Elevated plasma insulin is characteristic of T2D and also is proportional to body fat content [28]. In agreement with our data, investigators have noted that salivary insulin levels in obese children may be four or five times higher than in normal healthy weight children [29] and that salivary insulin correlates well with plasma levels at only slightly lower concentration [30]. Salivary insulin is linearly related to plasma insulin during the glucose tolerance test [11] and correlates well with plasma concentration after insulin injection [31]. Plasma insulin decreases in parallel with weight loss in obese children [32].

Salivary CRP concentration has also been found to correlate well with serum concentrations [24]. Association between salivary CRP and obesity has been reported in black South African children [33]. Human studies associate high levels of CRP with metabolic syndrome and T2D [34]. Reduced adiponectin is an independent risk factor for metabolic syndrome [35]. Both salivary adiponectin [24] and leptin [2] concentrations are highly correlated with plasma concentrations.

Since it is well-known that the concentration of proteins can vary significantly in response to stimulation, total protein is sometimes used to normalize concentration of specific salivary analytes [36]. The total protein content of salivary samples was reduced as salivary flow rate increased (Figure 2), however, neither total salivary protein nor salivary flow rate were significantly different between body weight categories (Figure 3) indicating that obesity did not affect salivary protein levels or salivary flow rate in this population. Investigation of a protein-normalized sample subset indicated that this procedure did not appreciably modify the diagnostic potential of salivary insulin, CRP, leptin or adiponectin in the samples collected from adolescent Kuwaiti children (Table 3).

The first level of the classification tree (CRP >219 pg/ml, Figure 4A), is associated with multiple inflammatory mediators (Table 4). The obese high salivary CRP (OC), represented the largest group of obese subjects (76.3%). The major differences among groups were elevated diastolic blood pressure, significantly reduced fitness and elevated leptin in the obese (OC) group. The non-obese high salivary CRP group (NC) had high levels of CRP, insulin, IL-6, IL-10, resistin, IL-1β and MMP-9 compared to the normal healthy group (NH).

The second level of the classification tree (CRP ≤219, insulin >128 pg/ml) also identified two groups of subjects (OI and NI), but these did not have elevated salivary inflammatory mediators but did have elevated insulin levels. The principal differences were that obese subjects had high systolic and diastolic blood pressures. Subjects with both low insulin and low CRP included a normal healthy group (NH) and an obese, ‘healthy’ group (OH). In this case, obese subjects were associated with elevated insulin, reduced adiponectin and reduced IL-10.

Comparison of these observations with those based on immunohistochemistry of obese adult adipose tissue [37] strongly suggests that the group designated as CLS+ (macrophage crown-like structure positive), found in 72% of obese adults, may coincide with the subjects designated as CRP >219 in our studies (76.3% of obese subjects). Our studies suggest, however, that non-obese cohorts with comparable biomarker levels could be at risk for development of immunometabolic complications of obesity.

The constellation of biomarkers that appear in saliva are likely a reflection of underlying pathology [38]–[40]. High concentrations of salivary CRP, myoglobin and MPO, for example appear after myocardial infarction [41]. Periodontal disease characteristically exhibits high levels of salivary IL-8 [42], MMP-9 and IL-1β [43]. Inflammatory bowel disease is associated with elevated salivary IL-6 and CRP [44]. We can now conclude that adolescent obesity is most commonly associated with high levels of salivary insulin and CRP.

## Conclusions

We have identified four salivary biomarkers in 10-year old subjects that change significantly with increasing obesity; insulin, CRP, adiponectin and leptin. The results of this study suggest that obesity may be characterized and classified by salivary biomarker concentrations. Use of these relatively non-invasive markers, particularly in longitudinal studies, to investigate development of metabolic diseases in children and evaluate therapeutic interventions could be used to evaluate preventative therapies.

## Supporting Information

Figure S1 **The concentration of all biomarkers in saliva supernatant tested by body weight category.** Values represent medians (center bar) +25^th^ percentile and −75^th^ percentile on a logarithmic axis for each category.
(TIF)

File S1 **File includes Tables S1–S3.** Table S1: Age, BMI, waist circumference and systolic blood pressure of 53 U.S. children (mean ± S.D) used to determine the saliva and plasma calibration curve (Figure 4B). Table S2: Saliva supernatant concentration, manufacturer’s stated assay sensitivity, assay precision and lowest assay standard of 20 biomarkers measured by multiplex assay. Three biomarkers, IL-10, leptin and ghrelin had median concentrations less than the assay sensitivity. Table S3: Concentration of sixteen cytokines in saliva supernatant of Kuwaiti children by body weight category and gender. Summary statistics are median, interquartile range (N subjects). Probability levels for overweight, obese and underweight were computed by Wilcoxon regression relative to normal healthy weight children.
(DOCX)

## References

[1] Zimmet P, Alberti KG, Kaufman F, Tajima N, Silink M, et al (2007) The metabolic syndrome in children and adolescents - an IDF consensus report. Pediatr Diabetes 8: 299–306. 17850473 10.1111/j.1399-5448.2007.00271.x

[2] Groschl M, Rauh M, Wagner R, Neuhuber W, Metzler M, et al (2001) Identification of leptin in human saliva. JClinEndocrinolMetab 86: 5234–5239. 10.1210/jcem.86.11.7998 11701683

[3] Gupta M, Chaturvedi R, Jain A (2013) Role of monocyte chemoattractant protein-1 (MCP-1) as an immune-diagnostic biomarker in the pathogenesis of chronic periodontal disease. Cytokine 61: 892–897. 23375122 10.1016/j.cyto.2012.12.012

[4] Gornowicz A, Bielawska A, Bielawski K, Grabowska SZ, Wojcicka A, et al (2012) Pro-inflammatory cytokines in saliva of adolescents with dental caries disease. Ann Agric Environ Med 19: 711–716. 23311795

[5] Teles RP, Likhari V, Socransky SS, Haffajee AD (2009) Salivary cytokine levels in subjects with chronic periodontitis and in periodontally healthy individuals: a cross-sectional study. J Periodontal Res 44: 411–417. 19210336 10.1111/j.1600-0765.2008.01119.x PMC2712869

[6] Sexton WM, Lin Y, Kryscio RJ, Dawson DR 3rd, Ebersole JL, et al (2011) Salivary biomarkers of periodontal disease in response to treatment. J Clin Periodontol 38: 434–441. 21480939 10.1111/j.1600-051X.2011.01706.x PMC3095429

[7] Kang EH, Lee YJ, Hyon JY, Yun PY, Song YW (2011) Salivary cytokine profiles in primary Sjogren’s syndrome differ from those in non-Sjogren sicca in terms of TNF-alpha levels and Th-1/Th-2 ratios. Clin Exp Rheumatol 29: 970–976. 22132900

[8] Arellano-Garcia ME, Hu S, Wang J, Henson B, Zhou H, et al (2008) Multiplexed immunobead-based assay for detection of oral cancer protein biomarkers in saliva. Oral Dis 14: 705–712. 19193200 10.1111/j.1601-0825.2008.01488.x PMC2675698

[9] Scheicher ME, Teixeira MM, Cunha FQ, Teixeira AL Jr, Filho JT, et al (2007) Eotaxin-2 in sputum cell culture to evaluate asthma inflammation. Eur Respir J 29: 489–495. 17079258 10.1183/09031936.00060205

[10] Ozcaka O, Nalbantsoy A, Buduneli N (2011) Interleukin-17 and interleukin-18 levels in saliva and plasma of patients with chronic periodontitis. J Periodontal Res 46: 592–598. 21635252 10.1111/j.1600-0765.2011.01377.x

[11] Marchetti P, Benzi L, Masoni A, Cecchetti P, Giannarelli R, et al (1986) Salivary insulin concentrations in type 2 (non-insulin-dependent) diabetic patients and obese non-diabetic subjects: relationship to changes in plasma insulin levels after an oral glucose load. Diabetologia 29: 695–698. 3542670 10.1007/BF00870278

[12] Aydin S (2007) A comparison of ghrelin, glucose, alpha-amylase and protein levels in saliva from diabetics. J Biochem Mol Biol 40: 29–35. 17244479 10.5483/bmbrep.2007.40.1.029

[13] Rai B, Kharb S, Jain R, Anand SC (2008) Biomarkers of periodontitis in oral fluids. J Oral Sci 50: 53–56. 18403884 10.2334/josnusd.50.53

[14] Shpitzer T, Hamzany Y, Bahar G, Feinmesser R, Savulescu D, et al (2009) Salivary analysis of oral cancer biomarkers. Br J Cancer 101: 1194–1198. 19789535 10.1038/sj.bjc.6605290 PMC2768098

[15] Tenovuo J, Lehtonen OP, Viikari J, Larjava H, Vilja P, et al (1986) Immunoglobulins and innate antimicrobial factors in whole saliva of patients with insulin-dependent diabetes mellitus. J Dent Res 65: 62–66. 3455700 10.1177/00220345860650011101

[16] Surdacka A, Ciezka E, Piorunska-Stolzmann M, Wender-Ozegowska E, Korybalska K, et al (2011) Relation of salivary antioxidant status and cytokine levels to clinical parameters of oral health in pregnant women with diabetes. Arch Oral Biol 56: 428–436. 21145038 10.1016/j.archoralbio.2010.11.005

[17] Janket SJ, Jones JA, Meurman JH, Baird AE, Van Dyke TE (2008) Oral infection, hyperglycemia, and endothelial dysfunction. Oral Surg Oral Med Oral Pathol Oral Radiol Endod 105: 173–179. 17905606 10.1016/j.tripleo.2007.06.027 PMC2574939

[18] Groschl M (2009) The physiological role of hormones in saliva. Bioessays 31: 843–852. 19554609 10.1002/bies.200900013

[19] Al Rashdan I, Al Nesef Y (2009) Prevalence of overweight, obesity, and metabolic syndrome among adult Kuwaitis: results from community-based national survey. Angiology 61: 42–48. 19398424 10.1177/0003319709333226

[20] Kahn SE, Zinman B, Haffner SM, O’Neill MC, Kravitz BG, et al (2006) Obesity is a major determinant of the association of C-reactive protein levels and the metabolic syndrome in type 2 diabetes. Diabetes 55: 2357–2364. 16873701 10.2337/db06-0116

[21] Rosa JS, Heydari S, Oliver SR, Flores RL, Pontello AM, et al (2011) Inflammatory cytokine profiles during exercise in obese, diabetic, and healthy children. J Clin Res Pediatr Endocrinol 3: 115–121. 21911323 10.4274/jcrpe.v3i3.23 PMC3184511

[22] Suriano K, Curran J, Byrne SM, Jones TW, Davis EA (2010) Fatness, Fitness, and Increased Cardiovascular Risk in Young Children. J Pediatr. 10.1016/j.jpeds.2010.04.042 20542285

[23] Ogden CL, Kuczmarski RJ, Flegal KM, Mei Z, Guo S, et al (2002) Centers for Disease Control and Prevention 2000 growth charts for the United States: improvements to the 1977 National Center for Health Statistics version. Pediatrics 109: 45–60. 11773541 10.1542/peds.109.1.45

[24] Browne RW, Kantarci A, LaMonte MJ, Andrews CA, Hovey KM, et al (2013) Performance of multiplex cytokine assays in serum and saliva among community-dwelling postmenopausal women. PLoS One 8: e59498. 23577067 10.1371/journal.pone.0059498 PMC3618114

[25] CDC (2012) Child and Teen BMI Calculator. Available: http://apps.nccd.cdc.gov/dnpabmi/.

[26] Hettmansperger TP, McKean JW (2012) Robust Nonparametric Statistical Methods Second Edition; Bunea F, Isham V, Keiding N, Louis T, Smith RL, et al., editors: CRC Press. Kindle Edition. 520 p.

[27] Shea S, Aymong E, Zybert P, Shamoon H, Tracy RP, et al (2003) Obesity, fasting plasma insulin, and C-reactive protein levels in healthy children. Obes Res 11: 95–103. 12529491 10.1038/oby.2003.15

[28] Bagdade JD, Bierman EL, Porte D Jr (1967) The significance of basal insulin levels in the evaluation of the insulin response to glucose in diabetic and nondiabetic subjects. J Clin Invest 46: 1549–1557. 6061732 10.1172/JCI105646 PMC292903

[29] Kamaryt J, Stejskal J, Mrskos A, Lavicky P (1989) [Insulin, glucose, proteins and amylase in the saliva of obese children]. Cesk Pediatr 44: 517–520. 2478304

[30] Pasic J, Pickup JC (1988) Salivary insulin in normal and type I diabetic subjects. Diabetes Care 11: 489–494. 3042316 10.2337/diacare.11.6.489

[31] Marchetti P, Giannarelli R, Masoni A, Cecchetti P, Di Carlo A, et al (1990) Salivary immunoreactive insulin concentrations are related to plasma free-insulin levels in insulin-treated diabetic patients. Diabete Metab 16: 16–20. 2185053

[32] Knip M, Nuutinen O (1993) Long-term effects of weight reduction on serum lipids and plasma insulin in obese children. Am J Clin Nutr 57: 490–493. 8460603 10.1093/ajcn/57.4.490

[33] Naidoo T, Konkol K, Biccard B, Dudose K, McKune AJ (2012) Elevated salivary C-reactive protein predicted by low cardio-respiratory fitness and being overweight in African children. Cardiovasc J Afr 23: 501–506. 23108518 10.5830/CVJA-2012-058 PMC3721867

[34] Shoelson SE, Lee J, Goldfine AB (2006) Inflammation and insulin resistance. J Clin Invest 116: 1793–1801. 16823477 10.1172/JCI29069 PMC1483173

[35] Renaldi O, Pramono B, Sinorita H, Purnomo LB, Asdie RH, et al (2009) Hypoadiponectinemia: a risk factor for metabolic syndrome. Acta Med Indones 41: 20–24. 19258676

[36] Lee JY, Chung JW, Kim YK, Chung SC, Kho HS (2007) Comparison of the composition of oral mucosal residual saliva with whole saliva. Oral Dis 13: 550–554. 17944671 10.1111/j.1601-0825.2006.01332.x

[37] Farb MG, Bigornia S, Mott M, Tanriverdi K, Morin KM, et al (2011) Reduced adipose tissue inflammation represents an intermediate cardiometabolic phenotype in obesity. J Am Coll Cardiol 58: 232–237. 21737012 10.1016/j.jacc.2011.01.051 PMC3132399

[38] Malamud D (2011) Saliva as a diagnostic fluid. Dent Clin North Am 55: 159–178. 21094724 10.1016/j.cden.2010.08.004 PMC3011946

[39] DeFuria J, Belkina AC, Jagannathan-Bogdan M, Snyder-Cappione J, Carr JD, et al (2013) B cells promote inflammation in obesity and type 2 diabetes through regulation of T-cell function and an inflammatory cytokine profile. Proc Natl Acad Sci U S A 110: 5133–5138. 23479618 10.1073/pnas.1215840110 PMC3612635

[40] Jagannathan-Bogdan M, McDonnell ME, Shin H, Rehman Q, Hasturk H, et al (2011) Elevated proinflammatory cytokine production by a skewed T cell compartment requires monocytes and promotes inflammation in type 2 diabetes. J Immunol 186: 1162–1172. 21169542 10.4049/jimmunol.1002615 PMC3089774

[41] Floriano PN, Christodoulides N, Miller CS, Ebersole JL, Spertus J, et al (2009) Use of saliva-based nano-biochip tests for acute myocardial infarction at the point of care: a feasibility study. Clin Chem 55: 1530–1538. 19556448 10.1373/clinchem.2008.117713 PMC7358990

[42] Rathnayake N, Akerman S, Klinge B, Lundegren N, Jansson H, et al (2013) Salivary biomarkers of oral health: a cross-sectional study. J Clin Periodontol 40: 140–147. 23174014 10.1111/jcpe.12038

[43] Kinney JS, Ramseier CA, Giannobile WV (2007) Oral fluid-based biomarkers of alveolar bone loss in periodontitis. Ann N Y Acad Sci 1098: 230–251. 17435132 10.1196/annals.1384.028 PMC2570328

[44] Aleksandra Nielsen A, Nederby Nielsen J, Schmedes A, Brandslund I, Hey H (2005) Saliva Interleukin-6 in patients with inflammatory bowel disease. Scand J Gastroenterol 40: 1444–1448. 16316893 10.1080/00365520510023774

