# Supplementary figures and images for: Metabolic Disease Risk in Children by Salivary Biomarker Analysis
Source: PLoS One. 2014 Jun 10;9(6):e98799. doi: 10.1371/journal.pone.0098799 (PMC4051609; doi:10.1371/journal.pone.0098799)

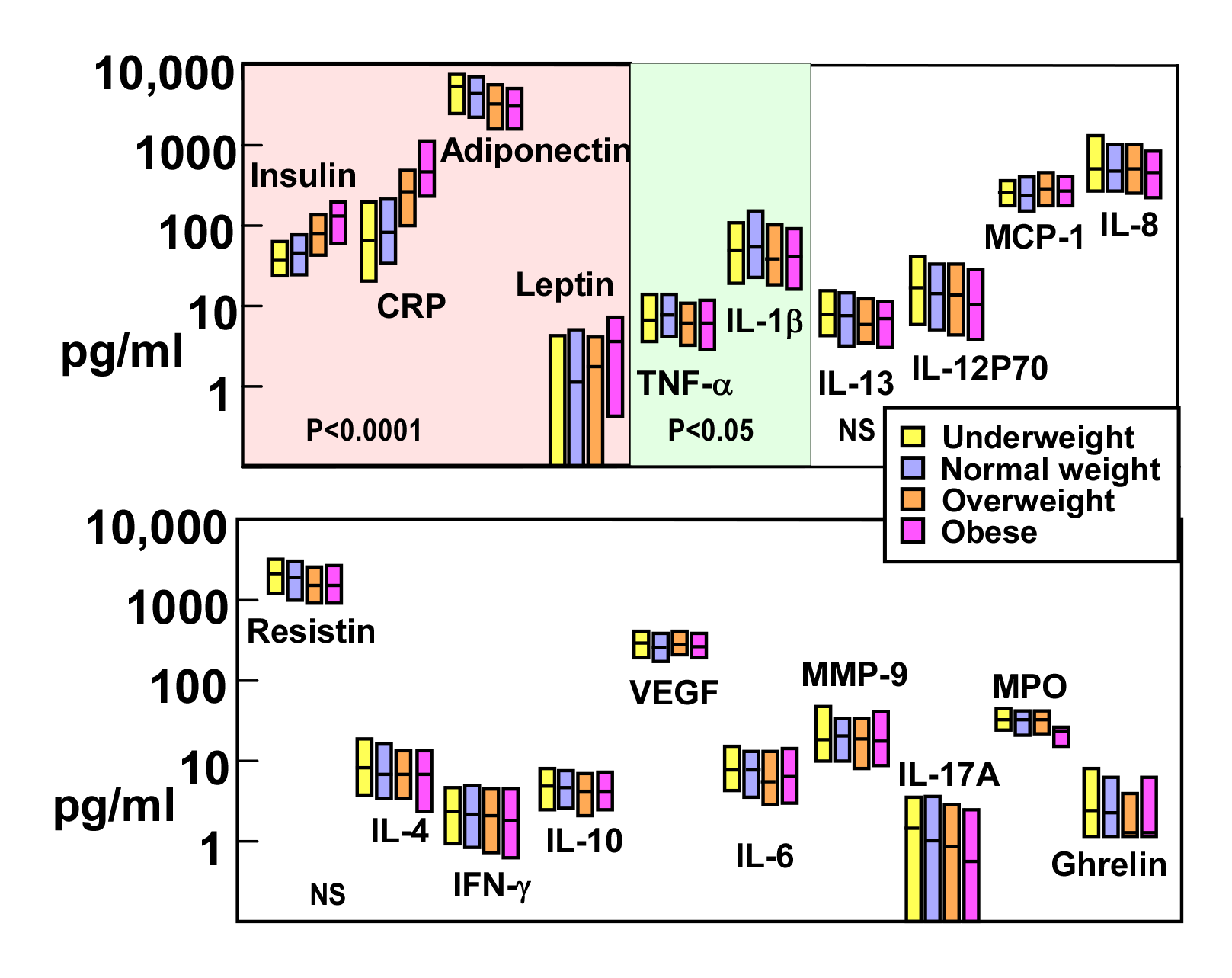

Supplement: Figure S1 — The concentration of all biomarkers in saliva supernatant tested by body weight category. Values represent medians (center bar) +25th percentile and −75th percentile on a logarithmic axis for each category. (TIF) [file pone.0098799.s001.tif]
