# Supplementary material for: Metabolic Disease Risk in Children by Salivary Biomarker Analysis
Source: PLoS One. 2014 Jun 10;9(6):e98799. doi: 10.1371/journal.pone.0098799 (PMC4051609; doi:10.1371/journal.pone.0098799)
Supplement: File S1 — File includes Tables S1–S3. Table S1: Age, BMI, waist circumference and systolic blood pressure of 53 U.S. children (mean ± S.D) used to determine the saliva and plasma calibration curve (Figure 4B). Table S2: Saliva supernatant concentration, manufacturer’s stated assay sensitivity, assay precision and lowest assay standard of 20 biomarkers measured by multiplex assay. Three biomarkers, IL-10, leptin and ghrelin had median concentrations less than the assay sensitivity. Table S3: Concentration of sixteen cytokines in saliva supernatant of Kuwaiti children by body weight category and gender. Summary statistics are median, interquartile range (N subjects). Probability levels for overweight, obese and underweight were computed by Wilcoxon regression relative to normal healthy weight children. (DOCX) [file pone.0098799.s002.docx]

Table S1

Table S2

Table S3
